# Mixed neuroendocrine non-neuroendocrine neoplasm of the gallbladder complicated by a pancreaticobiliary maljunction of a non-dilated biliary duct: A case report

**DOI:** 10.1097/MD.0000000000027336

**Published:** 2021-10-01

**Authors:** Kohei Wagatsuma, Kotaro Akita, Masayo Motoya, Yasutoshi Kimura, Shintaro Sugita, Takehiro Hirano, Yujiro Kawakami, Yasunao Numata, Keisuke Ishigami, Yoshiharu Masaki, Ayako Murota, Masahiro Shitani, Noriyuki Akutsu, Shigeru Sasaki, Hiroshi Nakase

**Affiliations:** a Department of Gastroenterology and Hepatology, Sapporo Medical University School of Medicine, Sapporo, Hokkaido, Japan; b Department of Surgery, Surgical Oncology and Science, Sapporo Medical University School of Medicine, Sapporo, Hokkaido, Japan; c Department of Surgical Pathology, Sapporo Medical University Hospital, Sapporo, Hokkaido, Japan.

**Keywords:** gallbladder, maljunction, mixed neuroendocrine non-neuroendocrine neoplasm, pancreaticobiliary, transdifferentiation

## Abstract

**Rationale:**

Mixed neuroendocrine non-neuroendocrine neoplasm (MiNEN) is a rare tumor. MiNEN of the gallbladder (GB) with pancreaticobiliary maljunction (PMJ) is extremely rare. The origin of MiNEN of the GB remains unknown; the biliary tract normally lacks neuroendocrine cells. MiNEN of the GB has a poor prognosis; because of its rarity, no treatment or management guidelines have been established yet.

**Patient concerns:**

A 47-year-old male presenting with right hypochondrial pain and malaise for 3 months was referred to our hospital for further management.

**Diagnosis:**

The neuron-specific enolase level was increased. Contrast-enhanced computed tomography revealed a mass of 70 mm in size with unclear boundaries in the liver. The GB was surrounded by this mass, narrowing the lumen of the GB. Many swollen lymph nodes were observed in the hepatoduodenal ligament. Endoscopic retrograde cholangiopancreatography revealed a PMJ with a non-dilated biliary duct. A percutaneous biopsy was performed on the liver mass, and the pathological findings were neuroendocrine carcinoma (NEC) (small cell type). We diagnosed a NEC of the GB, T3N1M0, stage IIIB (Union for International Cancer Control, 7th edition).

**Interventions:**

Because of advanced lymph node metastasis, we considered this tumor difficult to cure solely by surgical intervention. After initial chemotherapy consisting of cisplatin and irinotecan, a marked reduction in both tumor and lymph node sizes enabled conversion surgery. The pathological diagnosis of the resected tumor was MiNEN consisting of NEC and adenocarcinoma. The primary lesion was the adenocarcinoma occupying the luminal side of the GB. As a postsurgical treatment, the patient received additional irradiation therapy to the common hepatic duct and liver stump because of positive surgical margins.

**Outcomes:**

At 13 months postoperatively, computed tomography findings revealed the appearance of a hypervascular liver tumor, and laboratory data showed increased serum neuron-specific enolase levels. Chemotherapy was unsuccessful, leading to the death of the patient 36 months from the date of diagnosis.

**Lessons:**

There are several reports on the development of MiNEN of the GB. In our case, a PMJ-related adenocarcinoma of the GB transdifferentiated into NEC. Further accumulation of cases is necessary to establish a treatment strategy for MiNEN of the GB.

## 1 Introduction

Mixed neuroendocrine non-neuroendocrine neoplasm (MiNEN) is a rare tumor. In the World Health Organization classification of tumors of the digestive system (2010), mixed adenoneuroendocrine carcinoma was a term mainly reserved for combinations of adenocarcinoma and neuroendocrine carcinoma (NEC).^[1]^ In 2019, the World Health Organization renamed mixed adenoneuroendocrine carcinoma to MiNEN to also cover tumors other than adenocarcinoma and low-grade neuroendocrine tumors.^[2–4]^ In the pancreas and tubular gastrointestinal tract, MiNEN is characterized by the presence of at least 30% each of recognizable neuroendocrine and non-neuroendocrine components.^[5]^ However, such a cutoff is not defined for MiNEN in the gallbladder (GB). MiNENs have been reported at various sites of the gastrointestinal tract,^[6]^ but MiNEN of the GB is rare.^[7,8]^ In a recent study, MiNEN accounted for about 10% of GB cancers and about 2% of all hepatobiliary tract cancers.^[9]^ Recently published data suggest that MiNEN of the GB is probably more frequent than expected, as more than one-third of diagnosed GB NECs are associated with non-neuroendocrine components.^[10]^ The origin of MiNEN of the GB is still unknown because the biliary tract normally lacks neuroendocrine cells. MiNEN of the GB has a poor prognosis; partially due to its rarity and aggressive nature, it has no established treatment or management guidelines.

MiNEN of the GB with pancreaticobiliary maljunction (PMJ) is extremely rare.^[11–14]^ PMJ is a rare congenital anomaly and mostly prevalent in Asians, especially in the Japanese population. The prevalence rate of PMJ is 0.03% in Japan.^[15]^ PMJ is defined as the junction of the pancreatic and bile ducts outside the duodenal wall, causing pancreaticobiliary reflux and abnormally high levels of pancreatic enzymes in the bile. The biliary mucosal epithelium of patients with PMJ frequently shows mutations in genes such as *KRAS* and p53 tumor suppressor genes.^[16]^ Patients with PMJ have an increased incidence of biliary tract cancer,^[17,18]^ and PMJ, especially without bile duct dilation, is closely associated with GB mucosal hyperplasia and GB carcinogenesis.^[19]^

Here, we report a case of GB MiNEN with PMJ of a non-dilated biliary duct. Tubular adenocarcinoma components occupied the luminal side of the GB, and the invasive part was mainly composed of NEC (small cell carcinoma type). There have been no reports of such arrangements in the past, and it was suspected that the mechanism of MiNEN development involved PMJ-related adenocarcinoma of the GB transdifferentiating into neuroendocrine cancer.

## 2 Case presentation

A 47-year-old male experiencing right hypochondrial pain and malaise for 3 months was referred to our hospital for further management.

The patient had no significant past medical history. Physical examination revealed mild tenderness in the right hypochondrium, but the abdomen was soft, and a mass was not palpable. Blood cell count and renal function tests were within normal ranges. Serum bilirubin was also in the normal range whereas aspartate transaminase 158 U/L (normal range 11-39 U/L), alanine transaminase 225 U/L (normal range 5-40 U/L), alkaline phosphatase 523 U/L (normal range 110-370 U/L), gamma-glutamyltransferase 225 U/L (normal range 9-70 U/L), and amylase 130 U/L (normal range 37-120 ng/mL) were increased. Serum levels of tumor markers such as carcinoembryonic antigen, 19–9 carbohydrate antigen, and Duke Pancreas-2 were within normal ranges, but the neuron-specific enolase level was increased to 27.5 ng/mL (normal range 0-15.2 ng/mL).

**Figure 1:**
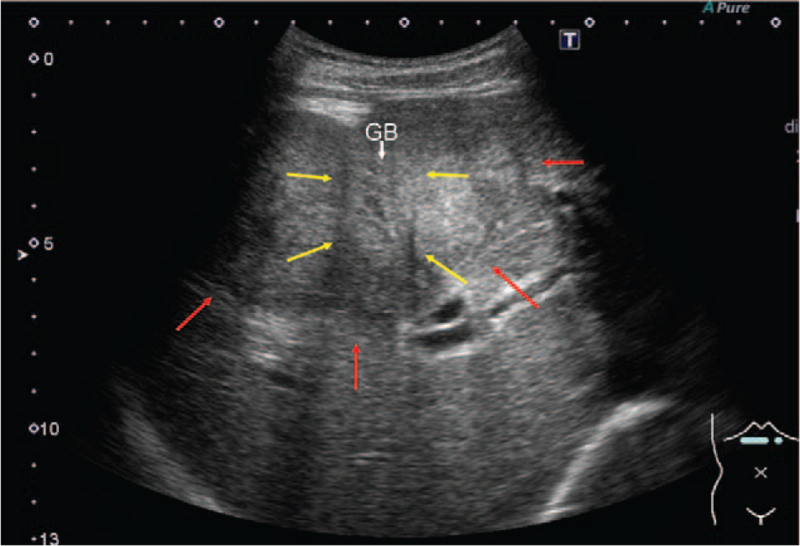
Abdominal ultrasonography findings at the time of diagnosis. Yellow arrow: the gallbladder with its narrowed lumen. Red arrow: a hyperechoic mass with an unclear border in the liver suspected of being connected to the gallbladder. GB = gallbladder.

**Figure 2:**
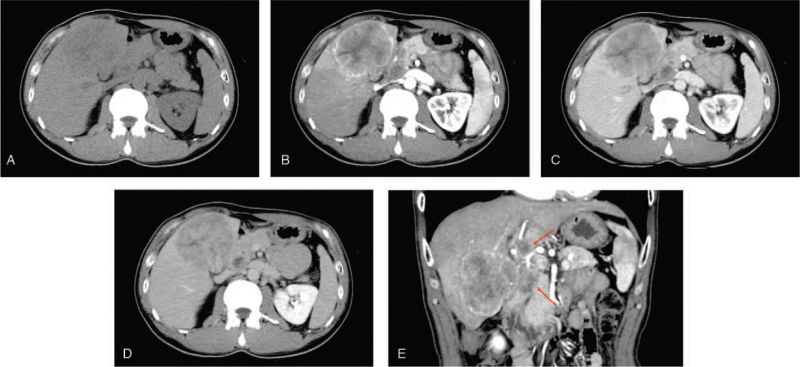
Contrast-enhanced computed tomography (CT) findings at the time of diagnosis. (A-D) Axial CT images in the (A) precontrast, (B) arterial, (C) portal venous, and (D) equilibrium phase. (E) Coronal contrast-enhanced CT image in the arterial phase. CT revealed a mass of 70 mm in size with indistinct margins occupying the anteromedial hepatic segments. Only the margin of this liver mass showed contrast enhancement. The gallbladder was surrounded by this liver mass, narrowing its lumen. Many swollen lymph nodes (red arrow) can be observed in the hepatoduodenal ligament.

**Figure 3:**
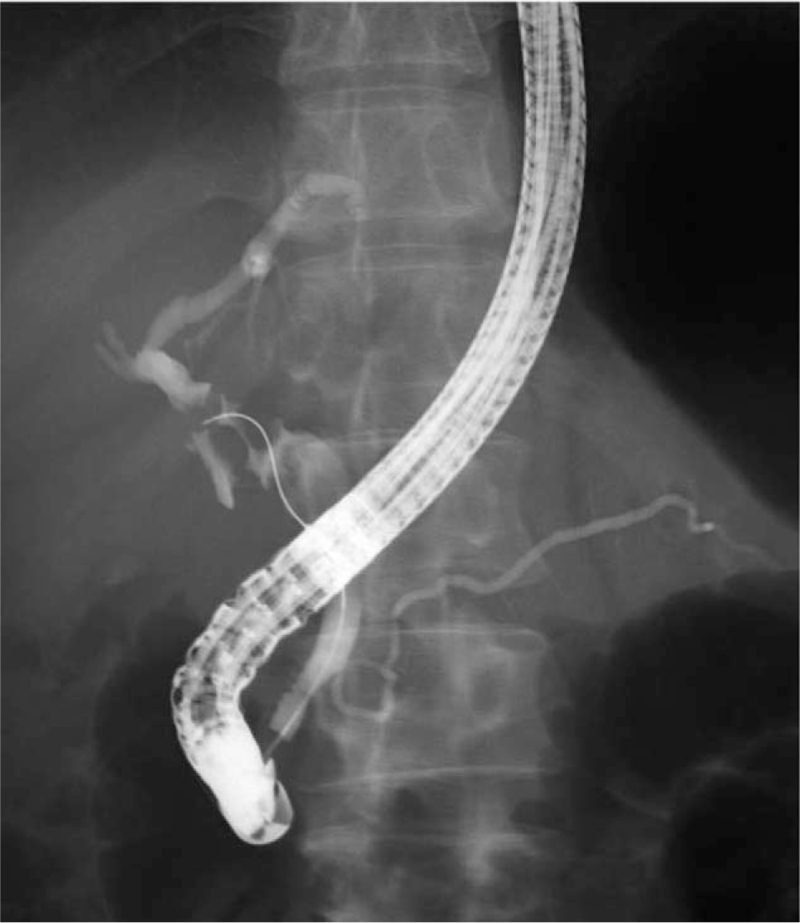
Endoscopic retrograde cholangiopancreatography (ERCP) findings at the time of diagnosis. An ERCP revealing a pancreaticobiliary maljunction of a non-dilated biliary duct. Multiple stenoses of the common bile duct caused by swollen lymph nodes can be observed.

Abdominal ultrasonography revealed a hyperechoic mass with an unclear border in the liver, suspected of being connected to the GB, which had a narrowed lumen (Fig. 1). Contrast-enhanced computed tomography revealed a mass of 70 mm in size with indistinct margins occupying the anteromedial hepatic segments. Only the margin of this liver mass showed contrast enhancement. The GB was surrounded by the liver mass, narrowing its lumen. Many swollen lymph nodes with suspected metastases were observed in the hepatoduodenal ligament (Fig. 2A-E). Endoscopic retrograde cholangiopancreatography revealed a PMJ with a non-dilated biliary duct. Considering multiple stenoses of the common bile duct caused by lymph node swelling, we placed a plastic stent in the common bile duct (Fig. 3). A percutaneous biopsy of the liver mass revealed an NEC (small cell type). Based on the finding that the GB tumor was contiguous with and surrounded by the liver tumor, we considered that the GB was the primary origin.

Thus, the patient was diagnosed with NEC of the GB, T3N1M0, stage IIIB (Union for International Cancer Control, 7th edition). Because of the advanced lymph node metastasis and the aggressive nature of biliary NECs, we considered the complete surgical removal of the tumor unsuitable and impractical for this patient. Therefore, we initiated chemotherapy consisting of cisplatin (60 mg/m^2^, intravenously on day 1) and irinotecan (60 mg/m^2^, intravenously on days 1, 8, and 15) repeated every 4 weeks.

**Figure 4:**
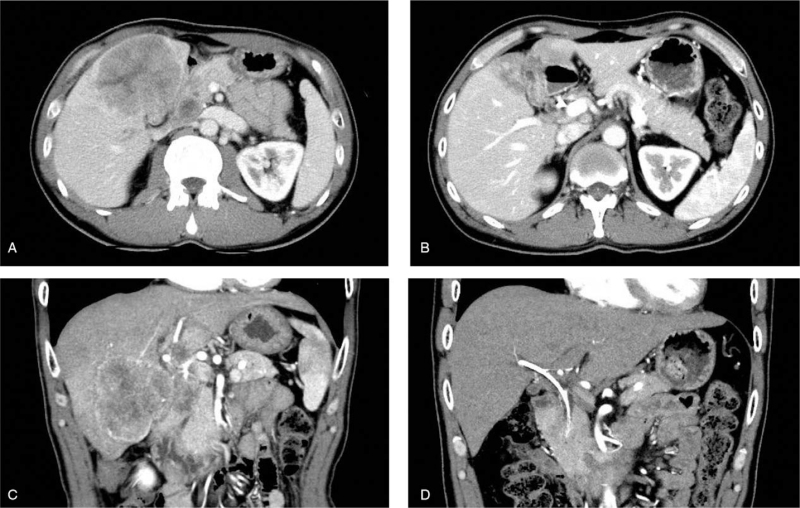
Contrast-enhanced computed tomography (CT) findings pre- and post-chemotherapy. (A, B) Axial portal venous phase. (C, D) Coronal arterial phase. (A, C) Pre-chemotherapy. (B, D) After 5 cycles of chemotherapy. (D) The plastic stent placed in the stenotic common bile duct is visible. Five courses of chemotherapy markedly reduced both liver tumor and lymph node sizes.

**Figure 5:**
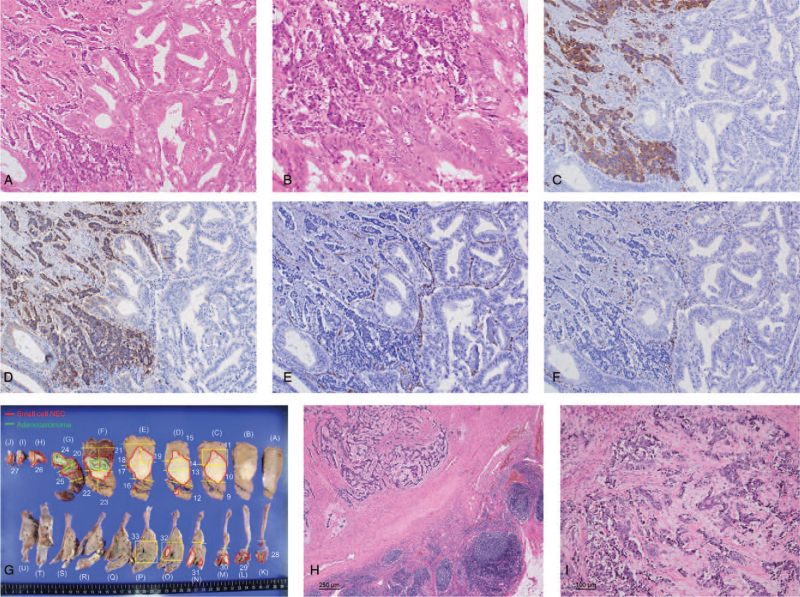
Pathological findings of the resected specimens. (A) Tubular adenocarcinoma and small cell carcinoma components can be observed. The 2 components are closely attached (hematoxylin and eosin staining, ×100). (B) A transition zone is visible between the tubular adenocarcinoma and small cell carcinoma components (hematoxylin and eosin staining, ×200). (C-F) Serial immunohistochemistry stainings (×100) adjacent to the sample shown in (A). (C) Synaptophysin-positive staining. (D) Chromogranin A-positive staining. (E) CD34-negative staining. (F) c-kit (CD117)-negative staining. (G) The distribution of the tubular adenocarcinoma and neuroendocrine carcinoma components is shown. The tubular adenocarcinoma component is predominant on the luminal side of the gallbladder and can be considered to be the primary lesion. By contrast, the gallbladder bed lesion invading the liver is mainly composed of neuroendocrine carcinoma (small cell type). Red: Neuroendocrine carcinoma. Green: Tubular adenocarcinoma. (H) Lymph node (hematoxylin and eosin staining, ×40). The main component metastasized to the lymph nodes is neuroendocrine carcinoma. (I) In the liver, foam cells and fibrotic lesions are found in the neuroendocrine cancer component (hematoxylin and eosin staining, ×100). NEC = neuroendocrine carcinoma.

Five courses of chemotherapy partially reduced the liver tumor (maximum diameter: from 76 to 43 mm) and eliminated the swollen lymph nodes (Fig. 4A-D). Subsequently, the patient underwent subtotal stomach-preserving pancreaticoduodenectomy and GB bed resection. Macroscopically, a grey-to-whitish, firm, and solid mass, measuring 4.5 × 3.5 cm in size, was located in the GB and invaded the GB bed. Histologically, the tumor was composed of tubular adenocarcinoma and small cell carcinoma components (Fig. 5A). The 2 components were closely attached via a transition zone (Fig. 5B). Pyloric gland metaplasia and intestinal epithelialization were not detected in the background mucosa. In immunohistochemical stainings, the small cell carcinoma component was positive for chromogranin A, synaptophysin, and CD56 (Fig. 5C, D). The Ki-67 labeling index was approximately 50%, and the average mitotic count was 3/10 high-power fields. However, the small cell carcinoma component was negative for CD34 and c-kit (CD117; Fig. 5E, F). Based on these findings, the pathological diagnosis of this component was an NEC (small cell type). The tubular adenocarcinoma component occupied the luminal side of the GB and was considered the primary lesion. On the other hand, the component invading the liver from the GB bed was mainly composed of NEC (Fig. 5G). There were lymph node metastases (8/34). Moreover, the NEC was the main component that invaded the extrahepatic bile duct and pancreas and metastasized to the lymph nodes (Fig. 5H, I). Therefore, we diagnosed this tumor as MiNEN of the GB, T4N1M0, stage IVA (Union for International Cancer Control, 7th edition).

As a postsurgical treatment, the patient received additional irradiation therapy to the common hepatic duct and liver stump because of positive surgical margins. At 13 months postoperatively, computed tomography findings revealed the appearance of a hypervascular liver tumor, and laboratory data showed increased serum neuron-specific enolase levels. The histological examination of biopsy specimens from the liver mass confirmed an NEC. We started the same chemotherapy regimen consisting of cisplatin and irinotecan (IP) because of the previous significant reduction in tumor size, but the tumor had progressed 5 months after chemotherapy initiation. We changed the chemotherapy from IP to cisplatin and etoposide (EP) therapy. However, the patient switched to palliative treatment 7 months after the start of the EP therapy due to the lack of significant effects. The patient died 36 months from the date of diagnosis because of multiple organ failure caused by the progression of MiNEN.

## 3 Discussion

We herein report the case of a 47-year-old man diagnosed with MiNEN of the GB. Based on the results of the liver biopsy, the initial diagnosis was NEC of the GB. Since several imaging findings indicated that complete surgical resection would be difficult, we started chemotherapy. Subsequently, the patient underwent surgical treatment because of the chemotherapy-induced drastic reduction in tumor size and lymph node swelling. The pathological results of the surgical specimens led to the diagnosis of MiNEN of the GB. In this case, an accompanying PMJ suggested its involvement in the development of the MiNEN. Moreover, the tubular adenocarcinoma occupied the luminal side of the GB, whereas the component invading the liver from the GB bed was mainly composed of NEC. Therefore, we hypothesized that the mechanism of MiNEN development was the transdifferentiation of adenocarcinoma to NEC during the invasion of the liver.

MiNEN is a rare tumor, and its prognosis remains unclear due to the heterogeneity of its components and the lack of an established treatment strategy. Moreover, the MiNEN pathogenesis remains elusive, although several reports regarding the mechanism of MiNEN development exist.^[20,21]^ MiNENs are histologically classified into 2 main types. One is the combined/biphasic type, in which neuroendocrine and non-neuroendocrine components such as adenocarcinoma proliferate within their respective territories. The other is the intermingling/amphicrine type in which neuroendocrine cells, non-neuroendocrine cells, and amphicrine cells, which combine differentiation characteristics of neuroendocrine and non-neuroendocrine cells, are spatially mixed while proliferating.

The origin of MiNEN of the GB remains unknown; the biliary tract normally lacks neuroendocrine cells. Three possibilities have been suggested for the histogenesis of neuroendocrine tumors in the GB. First, intestinal metaplasia of the GB mucosa occurs under chronic inflammatory conditions, and neuroendocrine cells contained in this metaplastic mucosa can be the origin of neuroendocrine tumors.^[13,22–25]^ Second, neuroendocrine tumors can directly arise from precursor stem cells having a multidirectional differentiation potential. These stem cells can be amphicrine cells.^[26–29]^ Third, a previously developed adenocarcinoma transdifferentiates into a neuroendocrine tumor.^[12,30]^

In this patient, the accompanying PMJ is of importance. Most cases of biliary tract cancers with PMJ are adenocarcinomas. It is recognized that PMJ contributes to various pathological changes in the GB, including mucosal hyperplasia, pyloric gland metaplasia, and metaplasia which can lead to the development of neuroendocrine tumors.^[31,32]^ In fact, there have been 4 reports of MiNEN in patients with PMJ (Table 1).^[11–14]^ These cases support the mechanism of MiNEN development in patients with PMJ outlined above. Five cases with Japanese patients, including our case, were reported. The mechanism of MiNEN development was considered to be transdifferentiation in 4 of 5 cases. So far, there had been no reports of MiNEN in which a tubular adenocarcinoma occupied the luminal side of the GB as in the current case. This was particularly important in considering the mechanism of MiNEN development. The invasive lesion from the GB bed to the liver mainly comprised NEC. In addition, the histological findings were indicative of a combined/biphasic type and the transdifferentiation from adenocarcinoma into NEC. On the other hand, pyloric gland metaplasia and intestinal epithelialization were not detected in the background mucosa, and the small cell carcinoma component was negative for CD34 and c-kit (CD117), which are markers of stem cells. Therefore, we hypothesized that in this case, the mechanism of MiNEN development was that PMJ-related adenocarcinoma of the GB transdifferentiated to NEC components during the invasion of the liver.

**Table 1:** Previously reported cases of mixed neuroendocrine non-neuroendocrine neoplasms of the gallbladder complicated by a pancreaticobiliary maljunction.

Author	Year	Age	Sex	Country	Components	Arrangement of non-neuroendocrine tumors	Mechanism of MiNEN development	Preoperative chemotherapy	Operation	Survival time after the operation	Reference
Oshiro H, et al	2008	55	F	Japan	SCNEC LCNEC AC	ND	Transdifferentiation	–	+	20 mo (alive)	^[13]^
Meguro N, et al	2014	54	F	Japan	LCNEC ICPN	ND	Transdifferentiation	–	+	24 mo (alive)	^[11]^
Michikawa Y, et al	2015	65	F	Japan	NEC AC	ND	Transdifferentiation	–	+	2 yrs (alive)	^[12]^
Kamei K, et al	2020	53	F	Japan	NEC AC	ND	ND	CDDP +GEM	+	27 mo (death)	^[14]^
Our study	2021	47	M	Japan	SCNEC AC	Occupied the luminal side of the gallbladder	Transdifferentiation	CDDP +CPT-11	+	30 mo (death)	-

AC = adenocarcinoma, CDDP = cisplatin, CPT-11 = irinotecan, GEM = gemcitabine, ICPN = intracystic papillary neoplasm, LCNEC = large cell neuroendocrine carcinoma, MiNEN = mixed neuroendocrine non-neuroendocrine neoplasm, ND = no data, NEC = neuroendocrine carcinoma, SCNEC = small cell neuroendocrine carcinoma.

In MiNEN, it is important to identify the predominant histology of advanced areas and metastatic lesions to be treated. When the neuroendocrine component is an NEC, this component is often more aggressive than the non-neuroendocrine component.^[33]^ However, patients with MiNEN of the GB lack early symptoms but present early vascular invasion and distant metastasis.^[7,8]^ Therefore, MiNEN of the GB is regarded as a disease with a poor prognosis. In the case presented here, IP chemotherapy led to a marked reduction in both liver tumor and metastatic lymph node sizes, enabling the surgical treatment of this patient. Despite several reports regarding successful surgery after extensive chemotherapy of MiNEN of the GB,^[12,34]^ the neuroendocrine tumors of the GB with local infiltration and lymph node metastases are at risk of recurrence and metastases after surgery, with a reported median survival of only 30.3 months.^[35]^

For MiNEN, there is no established chemotherapy. We initially selected IP therapy because some reports from Japan showed that IP therapy is superior to EP therapy in small cell lung cancer^[36]^ and in NEC of the gastrointestinal tract and hepatobiliary-pancreatic system.^[37]^ We considered that surgery was initially not indicated in this patient; however, the drastic effect of the initial chemotherapy led to conversion surgery. Despite the possibility that drug responses may vary depending on the combination of tumor components in MiNEN, the accumulation of cases will establish a chemotherapy strategy in the future.

In conclusion, there are various reports on the pathogenic mechanisms of MiNEN of the GB. In the current case, the mechanism of MiNEN development was that PMJ-related adenocarcinoma of the GB transdifferentiated to NEC components during the invasion of the liver. Further accumulation of cases is necessary to establish a treatment method for MiNEN of the GB.

## Acknowledgments

We would like to thank Editage (www.editage.com) for English language editing.

## Author contributions

**Conceptualization:** Kohei Wagatsuma, Kotaro Akita, Masayo Motoya, Yasutoshi Kimura, Shintaro Sugita, Hiroshi Nakase.

**Formal analysis:** Kohei Wagatsuma, Kotaro Akita, Masayo Motoya, Yasutoshi Kimura, Shintaro Sugita, Hiroshi Nakase.

**Funding acquisition:** Kohei Wagatsuma.

**Investigation:** Kohei Wagatsuma, Kotaro Akita, Masayo Motoya, Yasutoshi Kimura, Shintaro Sugita.

**Resources:** Kohei Wagatsuma, Kotaro Akita, Masayo Motoya, Yasutoshi Kimura, Shintaro Sugita, Takehiro Hirano, Yujiro Kawakami, Yasunao Numata, Keisuke Ishigami, Yoshiharu Masaki, Ayako Murota, Masahiro Shitani, Noriyuki Akutsu, Shigeru Sasaki.

**Supervision:** Hiroshi Nakase.

**Writing – original draft:** Kohei Wagatsuma.

**Writing – review & editing:** Yasutoshi Kimura, Shintaro Sugita, Hiroshi Nakase.
